# PyFF: A Fog-Based Flexible Architecture for Enabling Privacy-by-Design IoT-Based Communal Smart Environments †

**DOI:** 10.3390/s21113640

**Published:** 2021-05-24

**Authors:** Fatima Zohra Benhamida, Joan Navarro, Oihane Gómez-Carmona, Diego Casado-Mansilla, Diego López-de-Ipiña, Agustín Zaballos

**Affiliations:** 1 Laboratoire des Méthodes de Conception des Systèmes, Ecole Nationale Supérieure D’Informatique, Algiers 16309, Algeria; 2 DeustoTech, University of Deusto, 48007 Bilbao, Spain; oihane.gomezc@deusto.es (O.G.-C.); dcasado@deusto.es (D.C.-M.); dipina@deusto.es (D.L.-d.-I.); 3 Grup de Recerca en Internet Technologies & Storage (GRITS), La Salle—Universitat Ramon Llull, C/Quatre Camins, 30, 08022 Barcelona, Spain; joan.navarro@salle.url.edu (J.N.); agustin.zaballos@salle.url.edu (A.Z.)

**Keywords:** user involvement, fog computing, internet of things, privacy, flexibility, smart environments

## Abstract

The advent of the Internet of Things (IoT) and the massive growth of devices connected to the Internet are reshaping modern societies. However, human lifestyles are not evolving at the same pace as technology, which often derives into users’ reluctance and aversion. Although it is essential to consider user involvement/privacy while deploying IoT devices in a human-centric environment, current IoT architecture standards tend to neglect the degree of trust that humans require to adopt these technologies on a daily basis. In this regard, this paper proposes an architecture to enable privacy-by-design with human-in-the-loop IoT environments. In this regard, it first distills two IoT use-cases with high human interaction to analyze the interactions between human beings and IoT devices in an environment which had not previously been subject to the Internet of People principles.. Leveraging the lessons learned in these use-cases, the Privacy-enabling Fog-based and Flexible (PyFF) human-centric and human-aware architecture is proposed which brings together distributed and intelligent systems are brought together. PyFF aims to maintain end-users’ privacy by involving them in the whole data lifecycle, allowing them to decide which information can be monitored, where it can be computed and the appropriate feedback channels in accordance with human-in-the-loop principles.

## 1. Introduction

The Internet of Things (IoT)—committed to smartly connecting a deluge of digital assets deployed in users environments—is one of the main drivers of the digital transformation in modern societies [1]. The advent of the IoT has materialized the conception of a new interconnected world composed of new ubiquitous computing technologies. Several fields and domains ranging from education [2] to Industry 4.0 [3], including transportation [4], healthcare [5] and business [6], are exploiting the never-ending advances of IoT. Under this context, the overriding presence of technology can play a relevant role in addressing address new existing societal challenges [7] and bringing added value services in a way never imagined before. However, despite this continuous progress in smart services and technology, human beings seem to struggle to keep up with the pace of such digital achievements (e.g., smartphone adoption, use of social networks or e-administration services). On the one hand, the cultural divide, digital skills or economic inequality may hinder the equitable growth of these technologies [8]. On the other hand, human factors such as the apprehension about being tracked or privacy concerns relating to who may access the collected data can also be candidates to explain this issue [9,10]. This work focuses on the latter.

Generally speaking, people in modern societies are averse to be continuously surveyed (i.e., monitored) by a digital entity that they do not trust (i.e., up to what extent humans are confident with the data or service offered by a “thing” [11]), without knowing which data they are sharing [12]. This lack of trust continues to grow despite the efforts made by many initiatives on user and data privacy (e.g., GDPR (General Data Protection Regulation ) in Europe [13], CCPA (California Consumer Privacy Act) in USA [14] or LGPD (Lei Geral de Proteção de Dados (in English: General Data Protection Law)) in Brazil [15]). In addition, the lack of understanding about the behavior of these digital services (e.g., for a regular user, it is hard to grasp why a given IoT device has taken a certain decision) makes users lose their trust and perceived value toward them. Notwithstanding, the IoT paradigm should greatly contribute to boosting the involvement of human beings in new optimized services powered by technology and, hence, somehow minimize their reluctance [16].

Current IoT reference architectures [17], such as RAMI 4.0, IIRA, or even the IoT World Forum Reference Model, focus on specifying the hierarchical layers (also referred to as levels), information flows, functionalities and interoperability guidelines to design an IoT environment. However, the role of end-users is typically seen as a passive high-end interface rather than an embedded entity inside the whole data lifecycle (also referred to as human-in-the-loop [18]). Possibly, this design approach, together with the lack of standards for trustworthiness in the IoT [19], have led to the aforementioned trust concerns of IoT environments [11]. Note that these trust issues are more relevant than ever because of the current global COVID-19 situation and the measures taken by different countries to control the flows of people [20]. In the last months of 2020, society has witnessed important concerns raised over privacy involving the tracking strategies established to cope with the disease (i.e., technologies to track where people are, where they have been or what their disease status is) [21].

Therefore, the purpose of this paper is to propose a human-centric and human-aware (i.e., human-in-the-loop) IoT architecture where distributed and intelligent systems are brought together to foster user adoption and trustworthiness in IoT environments. In this regard, this work first proposes two different real-world use-cases to discuss the tangible challenges of enabling the digitization of user environments by means of IoT architectures, while considering user preferences, characteristics and behaviors. The findings and experiences collected from these two use-cases define the requirements of the proposed PyFF (Privacy-Fog-based Flexible): a user-oriented architecture for enabling privacy-by-design with human-in-the-loop IoT environments.

This work shows that understanding users and securing their privacy and including them into the data lifecycle, as done in PyFF, to make them aware of which data they are disclosing, is pivotal in the design and deployment of any IoT service that involves physical interaction [22]. In fact, PyFF is also envisaged as a first step to conceive Internet of People [23] architectures, where a shift from infrastructure-centric to human-centric environments is necessary. Although an extensive real-world deployment and evaluation of the PyFF architecture is still not available, the benefits of this approach are contextualized in the framework of a communal smart IoT environment: the digital transformation of a traditional office-based workplace. The selection of this particular use-case is conditioned by the additional difficulties it poses. Beyond the traditional privacy and security concerns that smart spaces need to face, smart workplaces propose additional threats. For example, privacy perception acquires new dimensions involving a social component, as these data can be associated with the image given to third parties or with the perception of productivity and work performance [24]. Additionally, due to the long hours that users (i.e., workers) spend in workplaces, this can be considered a strategic environment to address challenges such as user comfort and energy efficiency by means of IoT.

In essence, the contributions of this paper are twofold:

1. The PyFF architecture is proposed, which is conceived to transform digital environments while increasing energy efficiency, user comfort and maintaining users’ privacy. This architecture is derived from the analysis of two empirical studies (i.e., Smart Sustainable Coffee Machines and GreenSoul project) that are aimed to study user behaviors towards workplace digitization when it comes to automatizing energy-saving actions.
2. A multi-faceted qualitative comparison among the proposed PyFF architecture, GreenSoul and the Smart Sustainable Coffee Machines is presented. This comparison enables practitioners to assess the strengths and weaknesses of these three different IoT paradigms discussed in this work. In addition, these results can be taken as reference guidelines on how to convert a digital workplace into an appropriate setting to involve workers in decision making and motivate them towards more sustainable and healthier behaviors while promoting changes.

As an expanded version of our work in [25], we consider that the novelty of PyFF seeks to combine innovative data processing architectures, distributed intelligence processes and advanced immersive interaction interfaces between users and things to give place to user-aware (human-centred) IoT domains. This idea seeks to turn IoT environments into more efficient, trustworthy and acceptable scenarios for their users. Thus, we aim to transform the way users interact with their environment while promoting healthier behaviors or increasing levels of comfort for their occupants in return. PyFF offers a generalized version of fog-based privacy-aware architecture to use for any IoT-based smart environment.

To sum up, the proposal of the PyFF architecture, which puts humans in the loop within IoT environments, aims to contribute to making the Internet of People a reality and enable the conception of privacy-by-design IoT environments. The qualitative evaluation conducted in this paper shall guide developers and system architects to build reliable heterogeneous systems with regards to the data life cycle from the Edge to the Cloud.

The remainder of this paper is organized as follows. Section 2 details the two use-cases that inspired us to introduce and define the requirements of the new PyFF architecture: (1) the Smart Sustainable Coffee Machines project designed to test the effectiveness of persuasive technology to raise energy efficiency awareness in the mid an long term; and (2) the GreenSoul project that aimed at saving energy consumption in tertiary buildings engaging employees though bespoke ICT-based feedback. Section 3 depicts the PyFF architecture and discusses how it can be used to transform a digital workplace into a human-centric smart workplace. To better understand the functionality of the proposed architecture, an illustrative scenario is provided in Section 4 which showcases the flexibility and use of PyFF in a smart workplace scenario. Section 5 provides a qualitative comparison of the three IoT environments used in this paper. Section 6 compares our findings in the field of smart workplaces derived from the conception of PyFF with the related work. Finally, a discussion on the drivers and challenges and some conclusions are provided in Section 7.

## 2. Enabling the Digitization of User Environments by Means of IoT Architectures

To discuss the tangible challenges to enabling the digitalization of user environments, this section analyzes two already existing real-world uses-cases: (1) the Smart Sustainable Coffee Machine; and (2) the GreenSoul project. They are briefly introduced in the following.

- The Smart Sustainable Coffee Machines use-case [26] consists of instrumenting several capsule-based Coffee Machines in ten different work environments to provide them energy sensing and user-interaction capabilities. This scenario is aimed at measuring the importance of preserving user’s privacy when it comes to collecting sensitive data. The conducted experimental tests have led to a better understanding of the importance of user environment digitization and its side-effects. In fact, over-reliance on automation may bring undesired effects to pro-environmental behavior and reduce personal responsibility for action [27].
- The GreenSoul project use-case [28] consists of deploying IoT interactive artifacts to employees of six tertiary buildings across Europe (Austria, Greece, the UK and Spain) to enhance their awareness about energy consumption. The objective was to understand the new dynamics and discussions that these devices may bring in a communal context when they are deployed from scratch (e.g., the interaction with the device in the daily routine, the attachment or the confidence to the information they provide, emotions related to the IoT devices or their role as mediators of conversations among peers).

The analysis of these two use-cases combined with our previous work in [25] about boosting energy efficiency in smart workplaces, exhibits the key parameters that limit user involvement in IoT environments. Indeed, these use-cases have been used to collect new insights and issues on what IoT may bring to communal contexts. These insights have motivated the design requirements of the PyFF architecture.

### 2.1. Use-Case 1: Smart Sustainable Coffee Machines

The first use under analysis comes from an experimental intervention that took place over one year in 15 different sites with more than 100 users. This use-case was designed to assess the benefits of using IoT devices to increase users’ consciousness about energy consumption in a persuasive way. To this end, the Coffee Machines found in office environments were selected as the target IoT devices that would be used to persuade users to become more energy efficient (and aware) in the mid- and long-term. It is worth noting that the selection of the Coffee Machine for this experiment is not arbitrary. On the one hand, it is well-known that Coffee Machines are a commonplace asset in the majority of office-based working environments. On the other hand, due to the fact that Coffee Machines need to spend a considerable amount of energy maintaining the pump pressure and water heated, their power consumption can be higher than other A-class appliances such as modern refrigerators (i.e., A++), laptops, monitors or even ovens [29]. Full information and further details about the implementation of this experiment can be found in [26].

In the following, the main strategies to transform a regular appliance into an IoT device used for the sake of this use-case are summarized and the major findings on the user interaction with an IoT domain derived from this use-case are outlined.

#### 2.1.1. Preparation of the IoT Environment and Experiment Configuration

**Figure 1:**
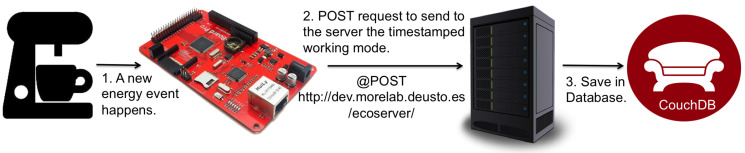
The energy consumption data flow from the Ethernet-based Arduino microcontroller board to the remote server where the data were stored for later processing and analysis [26].

As shown in Figure 1, embedded energy measurement equipment developed with an Arduino board was attached to the capsule-based Coffee Machines. The Arduino microcontroller resulted in a very convenient way to sense the energy consumption of the Coffee Machine—by means of an energy meter directly attached to its I/O ports—while, at the same time, providing a straightforward gateway to the Internet by means of its Ethernet port. This enabled the system itself to easily send energy consumption information to a remote server [30].

This layout enabled researchers to define three different experimental conditions related to how the users would be informed on energy awareness (i.e., how the IoT domain would interact with users): Automation, Persuasion and Web-based dashboard. Each experimental condition is detailed in the following:

1. Web-based dashboard: In this configuration, a website showing the energy consumption of each user from the coffee machine was developed. This enabled participants to monitor their own consumption and provide rational insights by means of showing historical data.
2. Persuasive feedback: This configuration combined subtle visual hints with ambient feedback provided in real-time to persuade the user to decide when the coffee machine should be turned off.
3. Automation: This configuration required no intervention from the user. In this way, the coffee machines decided themselves when was the best moment to shut down and did so accordingly. This was aimed at providing a notion of comfort for the users since they did not have to worry about switching the coffee machine off and on to save energy.

**Figure 2:**
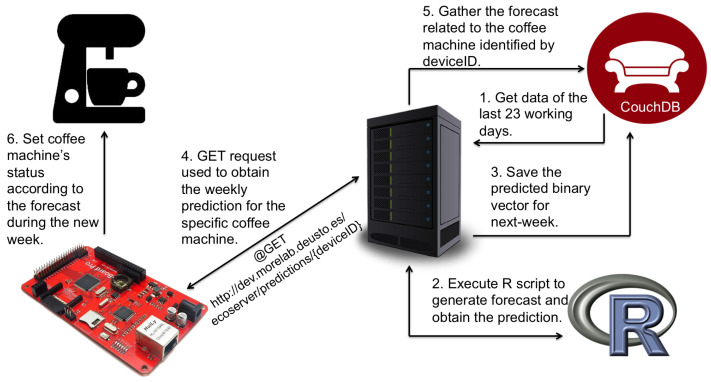
System architecture of the Coffee Machine use-case [26].

It is worth mentioning that the last two configurations (i.e., *Persuasive feedback* and *Automation*) used an Auto Regressive Integrated Moving Average (ARIMA) model (running on an external server rather than on the coffee machine itself due to the reduced storage and computing capabilities of the Arduino board) to statistically forecast the number of users who would use the appliance every hour of the day [31]. The final architecture to run the experiment is shown in Figure 2.

#### 2.1.2. Evaluation Procedure and Obtained Results

The evaluation procedure was based on structured questionnaires. These questionnaires aimed to obtain users information related to the socioeconomic profile of each participant to contextualize the experiment population; their pro-environmental attitudes [32] as well as their the pro-environmental readiness to change [33]; and their confidence in technology as a means to address environmental challenges. This information facilitated objectively assessing whether and up to what extent the users wanted to modify their pro-environmental behavior. It is worth noting that each participant enrolled in the experiment had to answer these questionnaires twice: once before the experiment and then after the experiment (i.e., 1 year later).

The obtained results and main lessons learned from this use-case are summarized in the following:

- Energy Consumption: After running the experiment with the IoT coffee machines, the energy consumption for the *Persuasive feedback* and *Automation* experimental conditions dropped by 44% and 14%, respectively. Surprisingly, no energy consumption reduction was observed in the *Web-based dashboard* experimental condition. Therefore, the following remarks can be inferred. First, it is possible to improve energy consumption of daily appliances. Second, human supervision can mitigate bias in statistical models (i.e., the *Persuasive feedback* condition saved more energy than the *Automation* one). Finally, persuasion is key to involving users (i.e., no changes where observed in the *Web-based dashboard* experimental condition)
- Questionnaires: After analyzing all the questionnaire data, it was found that the users of the *Automation* experimental condition were the ones who most distrusted the autonomous behavior of the coffee machine and, thus, felt skeptical that technology could be a driver for pro-environmental change. Additionally, after the experiment, this experimental group proved to be less likely to adopt attitudes to favor the environment. These findings are fairly well correlated with the work of Murtagh et al. [27], who found that automation impairs pro-environmental attitudes and undermines actions for personal responsibility. To sum up, the following remark can be inferred from the evidence above: autonomous appliances (e.g., the coffee machine in this use-case) may contribute to reduce the confidence and trust in technology. Therefore, user idiosyncrasy cannot be neglected when implementing automation in an IoT domain.
- Focus Groups: To further capture user feedback on this experiment, a set of focus groups was conducted. From them, the most relevant observation came from the users of the *Automation* experimental condition. Specifically, they complained about the fact that users were kept out the loop of the coffee machine operation. That is, it was not possible to intervene on the decision process that the coffee machine did to self shutdown. Users reported feelings of frustration when being unable to use the appliance at will—although they were aware that this was done to improve energy consumption.
  The main lesson learned from this situation is that users need to understand the behavior of an autonomous device in order to ensure a long-term effective coexistence.

Overall, the results obtained in this use-case shown an unexpected rebound effect associated to automation in IoT environments. To sum up, leaving the processes management—particularly, those ones related to energy efficiency—to automated entities (e.g., statistical and machine learning) may bring to averse phenomena: passivity to act in favor of the environment and widespread distrust on the suitability of technological solutions to address latent environmental issues.

### 2.2. Use-Case 2: GreenSoul Project

The second use-case, referred to as GreenSoul (GS) [28], was designed to optimize energy costs in tertiary buildings considering the individual profile of each user. Although this use-case is also targeted at energy consumption, GS takes a step forward from the Coffee Machine and considers user behavioral patterns in order to take/suggest actions.

Therefore, before giving personalized recommendations and/or subtle nudges on energy consumption, GS accurately monitored the operation of as many appliances as possible (e.g., monitors, heating, ventilation and air conditioning devices). In addition, GS considered the idiosyncrasy of each user in order to provide him/her with suitable, yet effective, feedback to reach the overall goal of increasing energy efficiency without neglecting privacy and comfort. Overall, GS took some of the lessons from the Coffee Machine use-case and proposed strengthening the engagement of end-users rather than to develop complex automation algorithms in order to obtain durable results.

In the following, the IoT infrastructure deployed on the buildings to optimize their energy consumption is summarized and the major findings on the user interaction with the IoT domain derived from this use-case are outlined.

#### 2.2.1. Preparation of the IoT Environment and Experiment Configuration

**Figure 3:**
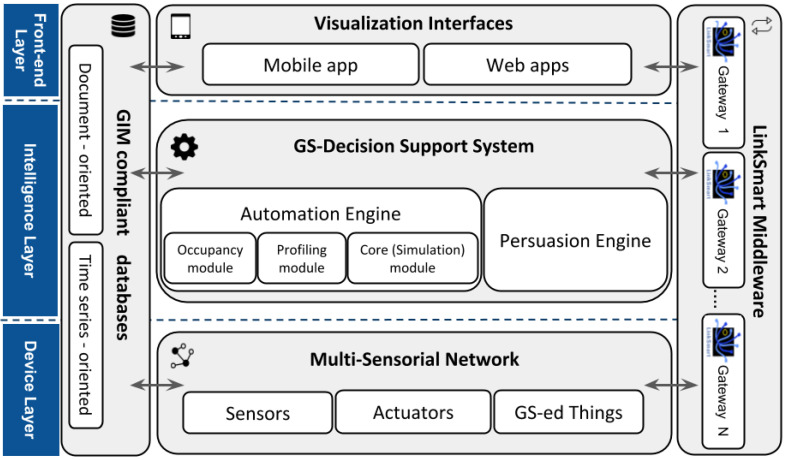
GreenSoul Reference Architecture [28].

A three-layered scheme following the physical building deployment and Edge Computing approach (Figure 3) was designed for the GS architecture: (1) the Device Layer; (2) the Building Layer; and (3) the Front-End Layer.

The *Device Layer*, the bottom part of the architecture, features the set of sensors that are considered relevant for data extraction and analysis; actuators that can be remotely controlled to assure that energy efficiency is achieved; and adaptors, which are new electronic devices connected to home or office appliances, of personal use (e.g., monitors, PCs, etc.) or collective use (e.g., printers, coffee-makers, outlets or power strips, etc.). Similarly to the smart coffee maker, the purpose of such adaptors was to optimize efficient usage of the mentioned appliances.

The *Building Layer* is responsible for giving value and meaning to the information retrieved. It consists of the GS-Decision Support System (GS-DSS) component, responsible for processing data and generating final operational recommendations at the Edge level.

Finally, the *Front-End Layer* features the components of the Visualization Interfaces that provide users access to mobile and web applications. With these interfaces, the GS platform will capture, store and manage energy-consumption data per device/user. Then, data are analyzed and displayed for educational and informative purposes.

The GS architecture benefits from flexibility in terms of: (1) enabling remote intelligent management of diverse remote devices (energy-meters and persuasive-ambient devices) always within the building; (2) applying persuasion techniques through GS-ed devices and mobile apps to eco-educate users both individually and at user-group level; and (3) providing device and environment decision-intelligence locally and at the Edge level to enhance the eco-friendliness profile of a given installation, where several common use devices are used by a group of users [26].

#### 2.2.2. Evaluation Procedure and Obtained Results

**Figure 4:**
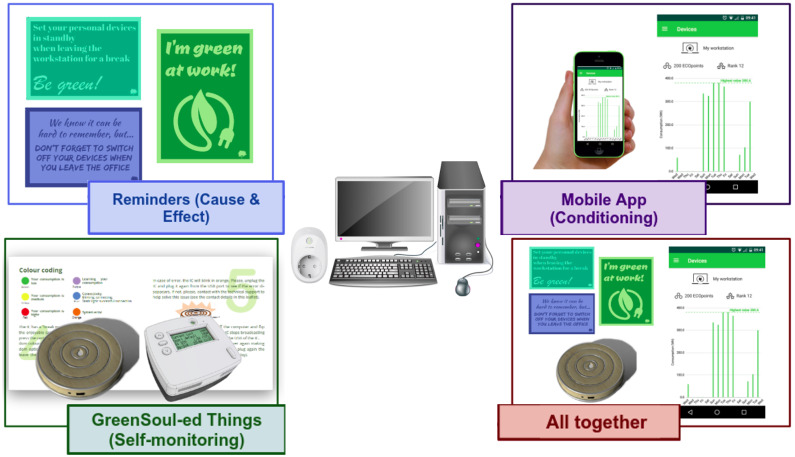
The GreenSoul Persuasion Treatments with the associated technology to deliver them (post-its, mobile app, physical devices and all the treatments together) [28].

The effectiveness of the overall GreenSoul system was tested by carrying out an intervention in six pilot buildings across Europe involving more than 350 people. Four different treatments combining three different persuasion principles through ICT were deployed (i.e., self-monitoring, cause–effect and conditioning). These treatments were delivered using different feedback channels: a custom-based interactive coaster that provided visual information about energy consumption (self-monitoring); a gamified mobile app with some automation features (conditioning); a series of analog signage in the form of post-its and posters with “green messages” (cause–effect), which can be considered as the control-treatment; and all three previous treatments together. Figure 4 illustrates each of them.

As with the smart coffee-maker intervention, this study was divided into two phases: individual and collective. During the individual phase, the primary objective was to foster the awareness and motivation of the participants in energy efficiency practices. Hence, the only individual information that was provided to end-users was regarding their performance with devices and appliances under their own control. In the second phase, we gave persuasive hints about how to reduce the energy consumption of electricity-powered devices not directly attached to the individual but more related to equipment of shared use (e.g., lighting, HVAC or common appliances).

Again, the overall GS solution was evaluated through a triangulation approach. To this aim, three different qualitative and quantitative sources were used: (1) pre–post validated surveys to assess energy awareness, motivations to change the behavior and main obstacles that hinder the adoption of energy practices in the workplace; (2) the energy consumption per user, per treatment and per building along the whole study; and (3) focus groups throughout all experimental phases to understand user motivations at each time, interventions pitfalls and other relevant matters.

The results emphasized the importance of understanding user profiles in both socioeconomic and behavioral terms to inform ICT-based campaigns to promote sustainable practices among employees. Related to privacy, automation and trust on systems and work-peers, we found that people trusted more in ICT interventions at the beginning, yet they simply presented cues of absentmindedness. Therefore, this suggests that providing frequent subtle feedback (i.e., reminders) to employees and tenants would contribute to helping users to remember green actions once they are aware of an energy-related problem. The GS intervention also shed light on the importance of understanding the level of confidence in technology if an ICT-based intervention to change the people’s behavior want to be applied. This finding was also relevant in the previous use-case. The pilots sites with higher levels of confidence in technology at the end of the intervention were found to be the ones with fewer barriers to behave energy efficiently. Finally, we also observed that high rates of confidence in technology and trust are correlated to a more actionable approach in favor of the environment.

To sum up, both use-cases stress the need to provide or maintain the confidence of end-users on technology if we want them to maintain their involvement on green actions suggested by ICT-interventions. This suggests the use of Fog/Edge Computing architectures to retain private data close to end-users while the whole internal process of computing the feedback is explained to them at any point.

### 2.3. Architecture Requirements for Enabling a Privacy-by-Design with Human-in-the-Loop IoT Environment

The results and experiences collected from the Smart Sustainable Coffee Machines and the GreenSoul project endorse the need to conceive a more flexible and privacy aware architectural solution. The most important insights derived from the analysis of these use-cases are summarized below:

- A fully-automated management system focused on energy efficiency seems to cause passivity among people to act in favor of the environment. In fact, users are not involved in actions which are automatically taken by the systems, and thus can hardly be influenced to adopt a good habit to help to reduce energy consumption.
- The automated system can also generate widespread distrust in the technology since it will discourage humans from taking the lead on their own actions.
- Users are often sensitive to sharing their data, resulting in users’ reluctance if the desired level of privacy is not respected. However, it is of paramount importance to sense as many data and monitor as many devices as possible to provide accurate recommendations (e.g., in health or energy-related scenarios) in order to increase end-users confidence.
- Since involving users to take actions in the smart environment is recommended, it is important to study their profiles in both socioeconomic and behavioral terms. This will help in defining the ICT intervention campaigns to communicate with each one accordingly and promote sustainable practices among users.

These insights allow us to define the following requirements that will guide the conception of the PyFF architecture:

1. Flexibility: The system must be able to provide different degrees of service at the same time according to the user profile and service to be delivered.
2. Privacy: The system must take into account the sensitivity of the data originated in the IoT environment, the service properties and user willingness to expose her/his associated data when exchanging and computing data over the IoT environment. Therefore, service performance shall be reduced, if necessary, to keep the desired privacy level.
3. Scalability: The system must provide for an ever-growing number of devices (and users) cohabiting and communicating among each others in the same IoT environment.
4. Including humans in the loop: The system must consider user preferences and behavior, which requires a shift from infrastructure-centric to human-centric [23] architectures. Therefore, users are no longer a high-end interface but a critical part on the whole information flow.
5. Data governance: The system must provide clear means to define which data will be exchanged, by whom and where they will be processed.

## 3. PyFF: A Privacy-Fog-Based Flexible Architecture

**Figure 5:**
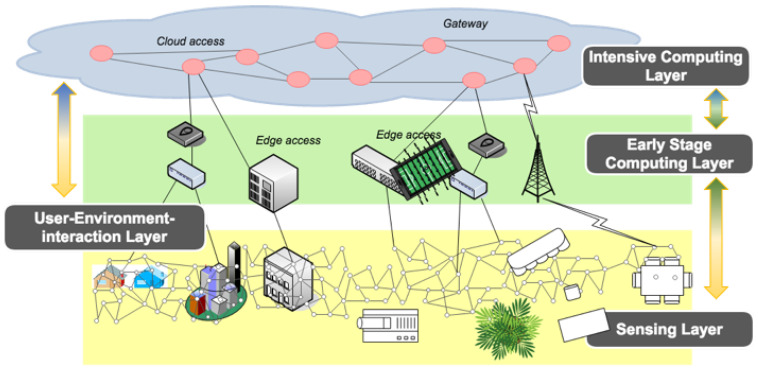
The proposed PyFF system architecture.

Driven by these reflections, this work proposes PyFF: a Privacy Fog-based Flexible architecture for IoT-based smart environments. PyFF features a distributed hierarchical system that takes advantage of the Fog Computing paradigm for enabling privacy-by-design with human-in-the-loop IoT environments. Specifically, PyFF is committed to: (1) collecting, storing and processing multi-modal data from low-cost devices in a scalable way; (2) providing several degrees of data privacy according to the user and application preferences; (3) hosting recommendation and forecasting distributed algorithms with variable computational cost; and (4) implementing ICT-based channels to communicate the concluded recommendations to users based on their profiles and preferences. Overall, based on a hierarchical design inspired by Fog Computing, we detail hereafter the PyFF system model and the functionalities of its layers. These layers are depicted in Figure 5.

From an architecture point of view, PyFF is compatible with existing well-known IoT architectures that, incidentally, are typically composed of three logical layers [34]: Perception layer (that could be mapped to the Sensing layer of PyFF), Network layer (that could be mapped to the Early Stage Computing Layer of PyFF) and Application layer (that could be mapped to the Intensive Computing layer of PyFF). However, existing architecture reference models (e.g., RAMI 4.0, IIRA, IoT-A and IEEE 2413-2019) focus on specific challenges (e.g., infrastructure data and connectivity, business usage implementation, interoperability and secure information exchange) and seem to neglect user involvement in the whole data lifecycle [35]. Therefore, PyFF aims to: (1) simplify the complexity of existing IoT reference models; and (2) enable privacy-by-design with human-in-the-loop IoT environments.

### 3.1. PyFF: System Model

The very first requirement that PyFF should meet—thoroughly learned from the GreenSoul use-case—is flexibility. The level of data privacy can change according to company policies and/or users preferences (e.g., users from the same company may have different privacy policies). Accordingly, the use-case of the Smart Sustainable Coffee Machines has stressed the relevance of providing user-adapted recommendations when using persuasive techniques to raise energy efficiency awareness. Therefore, PyFF must be able to adapt to the desired and dynamic levels of privacy, accuracy and automation. The flexibility of the proposed approach allows the user to interact with the system while iteratively personalizing it at any time. Thus, fine-grained control is given to the user, who has the power to modify and adjust the system behavior according to their privacy requirements and their current wiliness to be an active part of the process. This fine-grained control consists of specifying how “far” the associated data of users will go, that is which devices—and users—will store and/or process a certain datum for a given service. Such specification will be made by the user at service sign-up and epidemically propagated [36] to all the affected devices. This fine grained-control could be implemented by means of a declarative access control policy language such as XACML [37], which can be adapted to provide adaptive reasoning, as done in [38].

The Fog Computing nature of the proposed approach (see Figure 5) helps the system to be inherently flexible and enables it to integrate different technologies and standards with little effort, which makes it adaptable to any given scenario restrictions.

PyFF is composed of four main and flexible layers: (1) *Sensing Layer* is responsible for data collection; (2) *Early Stage Computing Layer* is represented by a Fog network used for local computation; (3) *Intensive Computing Layer* is deployed in a Cloud infrastructure and responsible for data aggregation, which is used to obtain more accurate recommendations; and (4) *User–Environment-interaction Layer* is used to optimize the interaction between the users and their surrounding smart devices while giving recommendations.

Such flexibility provides data processing, storage and networking scalable services between Cloud Computing infrastructures and IoT devices, generally located, but not exclusively, on the Edge of the network [39]. Indeed, the Fog Computing approach alleviates those fears related to sharing sensitive and private data on the Cloud by enabling users and applications to conduct intensive operations close to where the data were generated (i.e., Edge) and, thus, minimize the amount of information sent to the remote servers. This approach inherently increases data security since these data are kept inside the enterprise network and its firewalls, which can be best seen as privacy-by-design [40] enabler.

The four layers featured by PyFF are supervised by a Decision Support System (DSS) that, with the aid of the user, defines through intents the scope of every datum according to some rules such as privacy, presence or availability. This intent-based DSS is based on a previous work of the authors, the S${}^{3}$OiA framework [41]. Hence, PyFF can be considered a flexible architecture thanks to the fact that it can be decomposed into layers that can be added/removed depending on the system needs. The role and functionality of each layer is detailed in hereafter.

#### 3.1.1. Sensing Layer

Similar to submetering [42] in the electric field, the sensing layer is committed to collecting the greatest amount of data from the environment. It can be best seen as an IoT sub-domain where Internet-connected digital objects sense as many environmental variables as possible. For instance, a desktop computer can easily detect user presence, sitting posture and eye gaze/blinking by means of the built-in camera [43]. It can also infer user activity by counting keystrokes (or clicks on the mouse) during a period of time. Analogously, a smartphone can easily sense background noise, ambient light intensity or the amount of phone calls interrupting user’s activity. Additionally, other smart devices such as smart plugs, smart watches or smart speakers (digital assistants) can be easily reconfigured to report all the data that they seamlessly capture. Data communications in this Sensing Layer can be implemented by means of well-known protocols such as XMPP, MQTT or CoAP [44] since all sensed data will be later processed and matched to a certain behavior at the upper layers.

#### 3.1.2. Early Stage Computing Layer

Inspired from the Fog architecture, the Early Stage Computing Layer receives data from the Sensing Layer and conducts local non-intensive computations. From a data privacy point of view, this layer can be best seen as the frontier which sensible data shall not go beyond. In fact, as already seen in the GS use-case (see Section 2.2), several studies have shown that users, enterprises and stakeholders are keener to share and collaborate if those sensitive data are managed at the Edge of the network (i.e., fog) rather than outside of the premises [45].

Consequently, as long as the data privacy policies allow it, the Early Stage Computing Layer sends encrypted objects to the upper layer for strong recommendations or more sophisticated aggregated analytics. The latter requires greater computing power and more robust models.

Devices located at the Edge of the network can be typically identified as gateways, computers or local servers. Additionally, it is worth mentioning the situation in which the same physical device—due to its advanced sensing, computing and communication capabilities—can belong to the Sensing and Early Stage Computing Layers at the same time. This would be the case of the Arduino boards used in the Coffee Machines use-case (see Figure 1). One of these Arduino boards can locally decide (at the Early Stage Computing Layer) to turn on or off the coffee machines according to the current date and time, which would result in an immediate energy saving but may potentially lead to user dissatisfaction. However, before taking this decision, the Arduino board can check the overall energy consumption of the whole building (e.g., it might be empty) and decide—irrespective of the current date and time—to allow the user to have a cup of coffee. This is why this early stage layer transferring sensed data to the upper layer for more intensive computing and in exchange would obtain a richer and more accurate picture of the environment.

For a further explanation of the role of the Early Stage Computing Layer, imagine that a smart plug sends the power consumption of a heater. When the gateway detects that the heater has been working uninterruptedly for a specified number of hours, it might suggest to turn off the heater, which would result in energy saving—similar to the Smart Sustainable Coffee Machines use-case. In the upper layer (i.e., Intensive Computing Layer), the power consumption data will be correlated with other variables (e.g., office hours, office occupancy and ambient temperature) to make the recommendation stronger and, maybe, more widespread (e.g., in addition to the user, it could also trigger an alert to the staff in charge of facility management).

In addition, another example could be the situation where a potential camera is used to track users’ positions, and, thus, user privacy becomes of paramount importance. In this case, the proposal is to take an alternative approach by encrypting and sending to the following layer the user’s body/face edges and most notable features [43] instead of the whole video stream (as done in [46]). Note that this strategy intrinsically boosts worker’s privacy since it is guaranteed that: (1) the whole image stream cannot be reconstructed from the landmarks (i.e., no raw images are sent); and (2) no other environmental information of the user leaves the physical building. Additionally, the overall amount of data transferred to the communications network is greatly reduced, which increases the system performance.

Indeed, as data go from one layer to the next, the degree of data privacy is unavoidably reduced. Therefore, PyFF aims to move as few data as possible (following the principles of Cloud Computing [47]: move computation to data rather than moving data to computation) and, when the size of data or complexity of the computation associated to them makes it necessary for them to be sent to the next layer, data are encrypted (using a privacy scheme such as the one proposed in [48]).

#### 3.1.3. Intensive Computing and Storage Layer

Recent advances in machine learning require powerful computing platforms (e.g., GPUs) to run analysis and forecasting algorithms (e.g., those based on deep neural networks). This comes together with an eagerness of data. That is, these algorithms typically require large amounts of data to operate properly and provide accurate recommendations. For those applications/services that require these artificial intelligence algorithms, the modest features of devices deployed on the Edge network are not effective to appropriately handle such amount of data. Therefore, PyFF proposes a layer deployed in a Cloud infrastructure named as Intensive Computing and Storage Laye, which can be used at will whenever more computation and/or storage is needed (e.g., cloud bursting). Furthermore, this layer can also work as a complement for those applications where the processing capabilities are placed at the Early Stage Computing Layer. In those, inference tasks can be performed locally, where new data can be extracted, processed and converted into knowledge. Then, if the user allows their information to be externalized, learning models can be updated according to this extracted knowledge using the higher resources available at the Intensive Computing Layer.

At this point, the power of a Cloud Computing infrastructure is exploited by: (1) logging and aggregating all the collected data that reaches this layer—ideally, most of the data would reside on the lower layers; (2) using a computing-intensive Learning Classifier System able to build a set of user-readable rules (i.e., recommendations); and (3) forwarding these rules to the devices that have sensing but also acting capabilities from the Early Stage Computing Layer (i.e., User–Environment-interaction Layer). The recommendations resulting from this computing intensive data analysis will be mainly transmitted by means of the User–Environment-interaction Layer, which will be in charge of finding the best time/manner to deliver recommendations to the user (for instance, user’s presence must be guaranteed before making a recommendation), as previously learned with the Smart Sustainable Coffee Machines and GreenSoul use-cases. Note that the server used for the coffee machines use-case (see Figure 1) could be deployed in this layer.

#### 3.1.4. User–Environment-Interaction Layer

The availability of a large amount of data enables us to use this information to influence users and guide their actions towards more accurate and precise behaviors. For instance, it is better to recommend the user to switch off the light rather than telling him/her to reduce the energy consumption. For this reason, this layer oversees optimizing the interaction between the users and the devices by delivering contextualized feedback. This depends on when and how to interact with the users to effectively influence their behavior: on the one hand, by choosing the right recommendation mechanism (e.g., persuasive strategies based on personalized messages [26]), while, on the other hand, by selecting the right moment to provide the recommendations through anticipation (about-to-do moments) and reflection on action (just-in-time moments). The first one is based on anticipation, consisting of recognizing pre-action patterns that allow providing immediate interaction to redirect the activity through context-aware signals (lights, sounds or vibrations, among others). The second one consists on providing the user with all the information related to their behavior and performance, analyzing in depth patterns and changes over time and showing the possible consequences of this trend. Unlike the previous type of action, in this case, we seek to influence future habits through personal inquiry.

A second approach that PyFF also supports is related to closing the loop of interaction and allowing the users to not only receive information but also provide feedback to the system through intents [41]. Implementation wise, these intents are in line with the idea of the contemporary concept of human-in-the-loop [18] (i.e., human beings are the ones who guide an intelligent system as it learns) and with the way Amazon Alexa or other voice assistants are developed (available online: https://developer.amazon.com/en-US/docs/alexa/custom-skills/create-the-interaction-model-for-your-skill.html (accessed on 7 May 2021)). The intents and their associated utterances can be provided through multimodal interaction (e.g., tangible, voice-based or explicitly through a digital interface such as a web app or mobile app). These intents have to be propagated through the system to retrain and tailor the way and moment the feedback is provided according to the users’ criteria and needs. Hence, feedback and intents are two interwoven concepts towards personalization. The more the feedback from users is provided to the system, the sooner it will provide bespoke interaction in PyFF. The intents could be interpreted by the system through a rule base engine following the Rete Algorithm [49]. Some candidate implementations are Jess [50], CLIPS (available online: http://www.clipsrules.net/ (accessed on 7 May 2021)), pyKe (available online: http://pyke.sourceforge.net/index.html (accessed on 7 May 2021)) or Durable Rules (available online: https://github.com/jruizgit/rules (accessed on 7 May 2021)) which allow different programming languages for their implementation.

Finally, in certain applications/services, no recommendation to the end-user is required (see the *Automation* group in the Smart Sustainable Coffee Machines use-case). In this case, this layer could be removed/overlooked, which again shows the flexibility of the proposed system.

#### 3.1.5. Decision Support System

Since PyFF features a hierarchical heterogeneous architecture, a system orchestration is, hence, required to ensure communication and interoperability between the proposed four layers. PyFF integrates a Decision Support System (DSS) mainly based on middleware solutions for IoT-, Fog- and/or Cloud-based systems [51,52,53,54]. By investigating the work that Pore et al. [55] carried out on design issues for Fog and Edge middlewares, an approach using micro-services could be implemented to hold and orchestrate PyFF system. Indeed, some well-known Fog Computing frameworks such as Apache Edgent (available online: https://edgent.incubator.apache.org (accessed on 7 May 2021)) or Edgex Foundry (available online: https://docs.edgexfoundry.org/ (accessed on 7 May 2021)) use this paradigm that enables modular, scalable, secure and technology-agnostic applications [56]. In fact, the DSS with the aid of the user defines through intents the scope of every datum according to some rules. The list of rules to decide how to assign services and communicate between layers includes:

1. Privacy: Where users are enquired regarding their willingness in sharing sensitive data.
2. Accuracy: To decide where (i.e, Fog and/or Cloud) the computation (e.g., a recommendation) will take place.
3. User involvement: Where the system decides communication channels used to notify users based on their preferences and the multi-modal channels employed to assess how good or bad was the feedback received.

With the defined rules, the DSS covers communication and interaction between PyFF layers in order to decide: (1) which data to retrieve from physical devices; (2) how to protect data (anonymization, encryption, etc.); (3) which computation layer to address for recommendation (Fog or Cloud); (4) how to interfere with the environment to take actions based on computational results; and (5) how to communicate recommendations to users.

In essence, the main difference of PyFF with regards to other prior Fog/Edge architectures, systems and existing frameworks lies in the user involvement and the flexibility of the architecture to enable all the layers or just the most basic and functional ones. In other reviewed approaches, the end-user is mainly depicted as a bare consumer of the services provided by the architecture, usually in the top layer called “applications” or “marketplace”. However, PyFF provides a technology agnostic orchestration system able to put the user in the center of the decision making of what services offer and to what level of privacy they should be offered.

## 4. Illustrative Example: Smart Workplace

**Figure 6:**
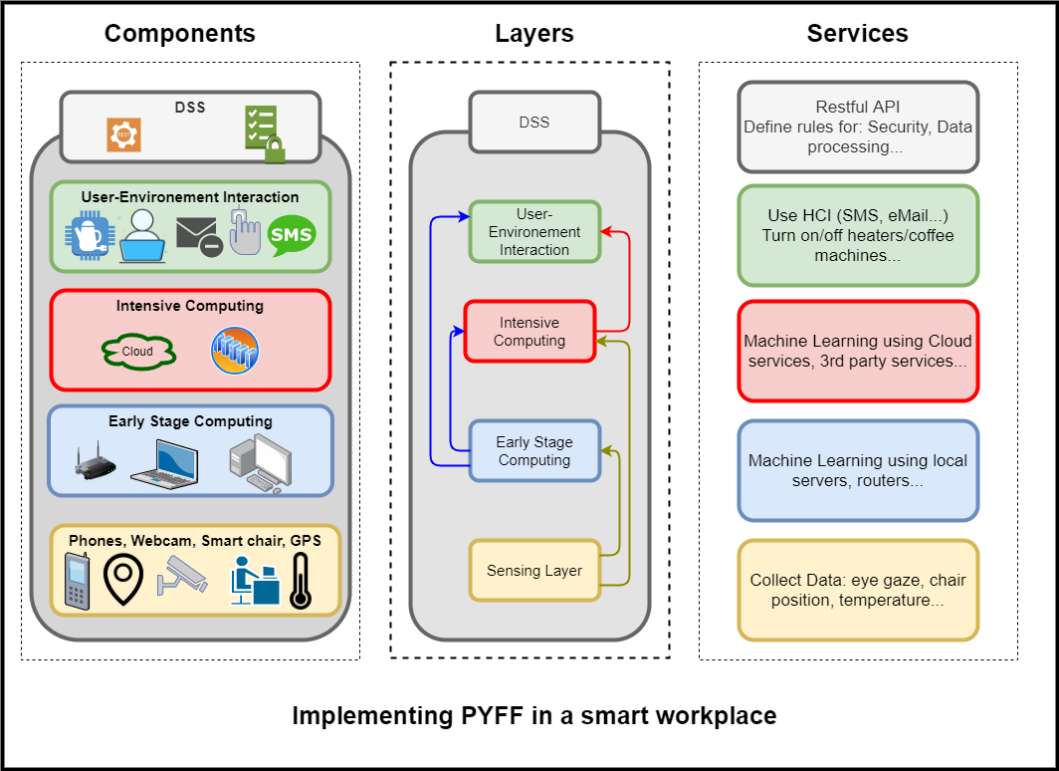
Abstraction of the PyFF architecture to address energy efficiency and user comfort in a smart workplace environment.

**Figure 7:**
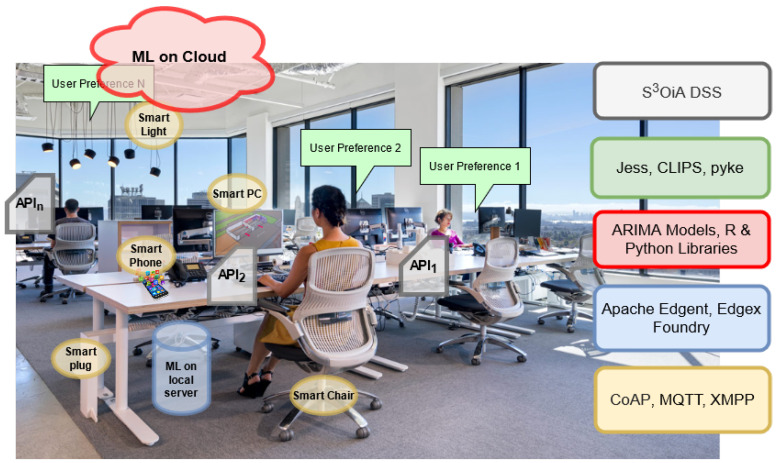
Implementation of the PyFF architecture in a smart workplace.

To better understand the functionality of the proposed architecture, an illustrative scenario to showcase the flexibility and use of PyFF is provided. This scenario is abstracted in Figure 6, while Figure 7 shows the mapping of the PyFF multi-layers architecture in a real-world environment. Let us consider an SME company that has several/shared offices for its workers and management. Every office, regardless of the employee category (i.e., blue or white collar) uses a set of standard devices (i.e., desktop computer with in-built camera, smartphone, smart plug, smart light and voice assistant) equipped with sensing capabilities in the workplace environment (yellow row in Figure 6 and yellow components in Figure 7).

On the one hand, the desktop computer of the office continuously monitors (i.e., Early Stage Computing Layer) the worker position and periodically triggers alerts when no significant movement is detected for long periods of time. This is aimed to improve the workers’ health conditions by reminding them to avoid sedentary attitudes (blue row in Figure 6 and blue server in Figure 7).

For those workers with no data privacy concerns (i.e., high-level staff may be averse to allow their sensed data to go away from the company), the face/body landmarks are sent to the Intensive Computing Layer (red row/cloud in Figure 6 and Figure 7) to precisely analyze the worker’s gaze, eye blinking and sitting posture. This layer sends back recommendations to the desktop (green row in Figure 6) in order to complement their local decisions (e.g., in addition to the “sedentary attitude” alert, another specific recommendation could be triggered: “perform neck exercises”). Note that, at this point, some users are taking advantage of the rich recommendations provided by the machine learning algorithms running at the Intensive Computing Layer (at the cost of assuming potential privacy leaks of the sensed data), while other users renounce these recommendations (at the price of keeping their sensed data safe). This flexibility is aimed at obtaining a larger user acceptance, as learned from the Smart Sustainable Coffee Machines and GreenSoul use-cases.

In this scenario, it is also worth considering the case in which a smartphone collects (Sensing Layer) data regarding ambient light intensity. When the smartphone detects an excess of ambient light (Early Stage Computing Layer), it triggers a notification for the user suggesting that s/he turns off the office light to reduce energy waste. Additionally, the ambient data sensed by the smartphone will again be cross-checked with data from other sources (e.g., it might be the case that the desktop screen is momentarily displaying bright images) in order to make a stronger recommendation (e.g., making an automatized phone call to the user). This is why, in some situations, the early stage layer needs to transfer sensed data to the upper layer for more intensive and correlated computing and global storage.

Similarly, the smart plug is continuously sending the power consumption to the same desktop application that locally monitors worker’s movements. This enables the system to autonomously infer behavioral status (via association rules [57]) from the user and his/her environment. For instance, with these rules, the system can assume—as early as at the Early Stage Computing Layer—that, if there is no movement and the fan is turned on (i.e., there is power consumption), the worker might have left and forgotten to turn off the fan and, thus, might decide to trigger a warning via the voice assistant, just in case the worker is still in the office. This inferred behavior must be further refined at the Intensive Computing Layer, where the power consumption of the smart plug will be correlated with the worker agenda to check whether the worker may be elsewhere and, thus, unilaterally decide to turn off the fan by means of the smart plug.

Finally, it is worth considering how the proposed system implicates users to get involved in these recommendations (to engage and leading them to a more responsible lifestyle) by means of the User–Environment-interaction Layer. In fact, workers are directly involved in changing their own habits in terms of energy waste. Users can configure the degree of privacy they want and through which interfaces (e.g., cell phone or email) they are willing to receive recommendations. Indeed, the system could be completely autonomous and, for instance, turn on and off devices accordingly, as done in the Smart Sustainable Coffee Machines use-case. However, in PyFF, we prefer in addition to implement a *user-unaware* energy efficient model, instill better intentions for workers. With this, we are avoiding users reluctance to technology as well as helping to tackle the root problem of energy consumption/waste by using those recommendations at a larger scale (i.e., at home, in public spaces and elsewhere).

Overall, with this example, it can be seen how the IoT architecture provided by PyFF can contribute to worker comfort and energy efficiency in a flexible and privacy-friendly, yet persuasive, way. In addition, as shown in Figure 6, the PyFF approach enables to add/remove layers according to the desired services or user constraints, which endorses the system flexibility. Indeed, one application may choose to use only the Early Stage Computing and the User–Environment-interaction Layers if all its users are reluctant to share their data. However, in the case of different data sensitivity preferences between users, both computing layers can be kept and only exclusively those data that meet the desired levels of privacy moved to the Cloud.

## 5. Qualitative Evaluation

We depict hereafter, a qualitative study to compare between both use-cases and the new PyFF architecture. The reason behind this cross validation is to demonstrate the improvement that PyFF brings in terms of flexible design for a smart workplace. Since PyFF has not been implemented yet, and since we conducted extensive experiments for both use-cases, Smart Sustainable Coffee Machines and GreenSoul, the comparison below is mainly based on the strategy of each architecture to enable a privacy-by-design with human-in-the-loop smart workplace. To this end, we define in Table 1 a set of metrics under three categories: (1) *Privacy*, to evaluate at up to what level the architecture respects privacy policies at corporate and/or employee level; (2) *Automation*, to assess the autonomy of the proposed system to offer the required optimization (e.g., energy efficiency) trading-off the degree of intrusiveness; (3) *Flexibility*, to estimate the possibility of re-adapting the design considering all potential parameters (physical components/architecture, ethical and privacy policies, size of data/network, etc.); and (4) *Deployment*, to assess the deployment efforts required to deploy it in a real-world environment.

To help read the qualitative comparison, we rank most factors varying from ++ (implemented/measured) to −− (not implemented). The ranking demonstrates how much the evaluation criterion was considered (or not) for each architecture. The example in Table 1 shows that data protection factor has been considered for both use-cases but relatively less than PyFF (anonymization schemes vs. privacy-based, user-centric scheme), with a + value for both GreenSoul and coffee Machine and ++ for PyFF. Seemingly, the disruption factor has clearly been neglected in the coffee machines because of the fully-automated (i.e., out-of-control) system which cost a—for its evaluation.

### 5.1. Privacy Metrics

Smart environments are challenging scenarios where technology is the primary way to collect data and obtain information about users. They must preserve users’ privacy and consider ethical concerns regarding personal data collection [58]. In Table 1, we evaluate the privacy through four main metrics: (1) Data Protection, i.e., what protocols are used to protect data; (2) Data usage, i.e., at what level we are disseminating data (Local/Edge, Cloud, etc.); (3) Homogeneity, i.e., whether we are using the same rule/protocol for every device/user in the application or not; and (4) Disruption/Intrusion, i.e., whether the new smart environment is being intrusive/disruptive to the user of not.

As demonstrated along the proposed two use-cases, users are more reluctant to be monitored in spaces that can be associated with their behaviors and habits (e.g., schedules and work performance in smart workplaces) [59]. In PyFF, privacy concerns are covered, ensuring the security of the data on every layer of the architecture, with special focus on the way sensitive information is processed and sent to the Cloud. Therefore, no unwanted personal data are made available. and, thus, the privacy of the users is preserved. In this regard, the Early Stage Computing Layer is introduced as an intermediate layer that offers local decisions based on data collected at the Sensing Layer and ensures sharing resources and services in the neighborhood of a network while enhancing their secrecy and availability. Nonetheless, in some applications, pre-processed data still need to be delivered to an upper layer with more computing and storage capabilities. To maintain the management requirements of the potentially sensitive information, the most critical point to consider on this layer is data privacy. Therefore, PyFF proposes to: (1) filter/transform personal data; and/or (2) encrypt data before sending them to the upper layer (i.e., Cloud services). Many existing security schemes can be used in this Fog-inspired architecture. For instance, SKES-Fog can be implemented as far as a smart environment architecture could be presented using domains, as suggested in [48]. Besides, data filtering or transformation allows deleting unnecessary data during the decision-making process (e.g., user’s identity). Later, the interaction layer will assign the anonymized data to its corresponding worker to send accurate recommendations (based on the decisions from the Intensive Computing and Early Stage Computing Layers) and receive feedback from them.

Furthermore, data need to be gathered without affecting users’ routine and minimizing their attention span, especially in workplaces. Thus, these systems need to be non-intrusive, creating an ecosystem surrounding the user that allows collecting data without any effect on his/her routine [60]. PyFF avoids intrusion and disruption by using digital devices already deployed in the environment or the users’ devices so that space is not over-instrumented with disruptive elements. In general terms, one of the strong points of a successful ICT initiative should be ensuring how the user interacts with technology, promoting its adherence while creating a sense of confidence and trust. While comparing PyFF architectural approach to the ones in the Smart Sustainable Coffee Machines and GreenSoul use-cases, we found both use-cases relatively intrusive. GreenSoul requires an amount of new deployed devices (which causes over-instrumentation in the smart environment) while Coffee Machine makes the system fully automatic which causes user’s reluctance. Since privacy is strongly based on the level of users’ adherence to sharing data and/or being instrumented with smart devices, PyFF enables privacy-by-design [61] with a heterogeneous scheme. With this, users have the choice of subscribing to the level of privacy they feel comfortable with (e.g., sharing data/identity, selecting a set of smart devices to collect data from, etc.) and update it according to the context or their current attitude towards the system. However, both use-cases implement one single protocol for all users and devices which make them less adaptive to users’ preferences and behaviors change on the run.

### 5.2. Automation Metrics

Designing a smart environment requires building autonomous processes to collect data, analyze information and make decisions. In the qualitative comparison, four metrics are defined to evaluate Automation in PyFF: (1) how much *user involvement* is respected; (2) what the level of *Recommendation accuracy* (i.e., intensive/early stage computation based on Cloud/Fog) is; (3) *ICT/HCI*, i.e., how the system interacts with users (Communication channels); and (4) if the system offers *Real-Time* services.

As concluded from the proposed use-cases, it remains important to communicate with the users during any actions/recommendations issued from the automation process. In fact, there is a risk of losing users’ trust and adherence in technology, while making the architecture totally automated (as in the Smart Sustainable Coffee Machines use-case). For this reason, PyFF was designed from a human-centered perspective to promote new habits in smart environments by considering the role of the user as a key factor in bringing changes. The basis of the change-management process is the way the information is used as an awareness mechanism and how this information is provided to the workers. In particular, information needs to be delivered effectively and digital feedback is an appropriate way to influence in the receiver [62]. In PyFF, the role of the user is boosted by the User–Environment-interaction Layer, in charge of optimizing the interaction between the users and the system through contextualized feedback [26] and privacy-based user intentions [41]. The former pursues involving the workers in the smart process and influencing their behavior through the application of technological persuasion techniques that increase their engagement and motivation. The latter allows the user to express the data a user wants to preserve and a set of requirements which have to be accomplished to this endeavor. Thus, the user will always be able to supervise the whole procedure in a reliable and understandable manner. This human-in-the-loop approach augments human interactions, making them part of the information retrieval, understanding and processing [63,64]. Thanks to this layer, Cloud services and humans in the loop transparently interact with each other, allowing a more secure and confident data exchange. The User–Environment-interaction Layer involves users in the process of promoting sustainable behaviors and, thus, encourages them to have confidence on a layered architecture that seeks to ensure the security, privacy and trust. This provides an adaptable interaction that can be dynamically adapted to different contexts and user preferences and, ultimately, allows users to educate the system (and reciprocally help the system educate the users) rather than relying exclusively on what the system decides for them.

### 5.3. Flexibility Metrics

An acceptable way to reach flexibility in a layer-based architecture is: (1) to offer *Adaptive Reasoning* by adding/removing layers in every application accordingly; (2) to implement *Context-based* protocols by offering a solution for any application domain (instead of only smart workplaces as in previous use-cases); and (3) to define a *scalable* solution that easily re-adapt to the size of network, devices and users (see Table 1).

The PyFF adaptive reasoning feature offers the possibility to add/remove one or more layers depending on the system needs (recall that we propose four layers). According to the service complexity, this can be implemented in the user registration process of the service by including a semantic reasoner or a simple questionnaire (e.g., “would you be comfortable with device X having access to your datum Y?”). The output of this module will be the privacy and behavioral rules that will constrain the scope of the service delivered to each user.

The addition and removal of PyFF layers is shown in the following examples. Let us first consider a use-case about a top-confidential work environment (i.e., military field): here, Cloud services can easily be excluded by removing the “Intensive Computing layer”, which may result in a reduced performance as long as the low layer devices lack from the required storage and computing capabilities to deliver service. Inversely, in a smart farm environment [65], where we need very accurate recommendations by aggregating data from all distributed lands (i.e., farms), and where privacy is not a big issue, there will be no need for the “Early Stage Computing Layer”. In addition, the role of the User–Environment-interaction Layer will be limited to communicate decisions to the user (i.e., farmer) without any suggestions of taking actions (because the goal behind the system is to remotely monitor the fields using deployed smart devices). These two examples—different from the smart workplace scenario—show that PyFF is a context-sensitive solution where its architecture can be generalized to a larger spectrum of use. Even though in this paper we focus on the energy-efficiency and users well-being in a smart workplace environment as an illustrative example, PyFF architecture is based on decoupling elementary services in any system (physical devices, privacy and computation rules, real-time and accuracy, HCI, etc.).

### 5.4. Deployment

When the size of IoT-based environments grows in terms of devices, the deployment and maintenance of their systems becomes relevant and intensive. It is very common to find IoT domains composed of heterogeneous and non-standardized devices, which makes them hard to deploy (e.g., individual configurations required) and maintain (e.g., when the system fails it is hard to find and isolate the faulty device).

Additionally, when the number of devices grows, the system may degrade its performance due to the communication overhead between devices and a lack of a scalable backbone. In this regard, the hierarchical approach featured by PyFF relies on Fog and Cloud Computing to alleviate the scalability issues emerged when facing a large number of IoT devices.

Furthermore, the distributed nature of PyFF makes it very robust against faulty IoT devices. These devices are known to be fault prone for several reasons (e.g., lack of reliable power sources, continuous exposition to harsh environments, etc.). In the likely case of a faulty IoT device, PyFF would be able to: (1) trace the source of the fault (i.e., the Intensive Computing Layer would identify non-coherent values compared to other sources or the Early Stage computing layer would receive very different values compared to its historic records); (2) isolate and ignore the faulty device (i.e., conducting a top-down analysis of the information flow along the hierarchical architecture); and (3) report to the user that a device is faulty (i.e., using the User–Environment-interaction Layer). Hence, the source of the events can be traced naturally.

## 6. Related Work

As shown above, technological advancements are starting to accelerate the evolution of future smart environments. Now, this concept goes much further than implementing technology to achieve this digital transformation and points to creating interactive spaces where people and technology collaborate. Under this vision, smart environments sense the physical world, give meaning to the obtained information and trigger suitable reactions to transform human lifestyles. As a consequence, the Internet of Things (IoT) can enhance health [66], wellness [67] or promote sustainable practices [68] in domains such as the city [69] or the workplace [70]. The latter is a good example of how human and machine intelligence can collaborate. Indeed, the inherent nature of these spaces, where an average employee spends a substantial part of her daily routine, involves that the habits and behaviors performed in the workplace play a key role in every individual and the society.

Thus, workplaces can be seen as ideal scenarios to guide workers towards new lifestyles that are extended beyond their workday [71]. Linking the workplace with health promotion and energy-related matters lead to the development of a sustainable working environment that increases awareness through healthier and more sustainable behaviors [72]. In particular, they can contribute to a more environmentally friendly energy management [73] and cover the lack of awareness of the individual about the impact these habits on their health [74]. For example, a work environment augmented with IoT can detect and classify unhealthy habits such as bad postures or sedentary habits and notify those harmful practices to end-users. Moreover, it can assist the user towards energy-awareness and to attain sustainable changes in the mid and long term.

A key factor when designing and implementing programs to promote new habits in the workplace is to study specific methods to identify which are the main problems and then to carry out useful strategies to solve them [75]. In this regard, ubiquitous technology can be used, firstly, to identify the unhealthy and unsustainable behaviors that are executed in these spaces and, secondly, to correct the inadequate practices that are recognized. Transforming the quality of the workplace experience implies monitoring which habits need to be changed and providing information about the consequences of these habits. Technology-based solutions allow us to physically or digitally interact with our surroundings to obtain data that can be transformed into information and, in the end, knowledge about the daily routines of the workers. Based on this knowledge, context-aware guidance can be provided to influence the users and change their behaviors. Thus, technology-based solutions can be considered appropriate drivers to promote wellness and energy awareness in the workplace.

Several attempts have been made to design enhanced workplaces [76] through the adoption of the Information and Communication Technologies (ICTs). From occupational risk assessment and ensuring safety in the workplaces [77], different solutions are proposed to reach large audiences and help them to prevent indirect risks associated with these spaces and bring energy awareness to their routine. For the former, occupational health and promoting more active behaviors in the workplace stand out as one of the most addressed concerns. In this direction, Taylor et al. reviewed the existing literature addressing interventions designed to reduce sitting time and the role of the organizational culture [78]. The obtained results coincide with the ones presented by Stephenson et al. [79], who concluded that interventions using a computer and mobile and wearable technologies can be useful in reducing these behaviors. The PEROSH initiative [80] studied how wearable devices could be part of wellness promotion interventions. It elaborated a decision support framework for selecting useful sensors and proper data collection strategies for avoiding sedentary behaviors neglecting data privacy issues. In the same way, Jimenez et al. [81] presented some guidelines to promote workplace health by using electronic and mobile health tools to provide easier administration for campaign proposers while considering data privacy from a technical and psychological points of view. However, no specific ICT architectures have been proposed to conduct these processes. Other works have approached wellness interventions through digital technologies and have also been proposed for reducing sedentary behaviors [82] as well as to increase energy expenditure and promote more active periods [83,84].

Commercial solutions such as Comfy (available online: https://www.comfyapp.com/ (accessed on 7 May 2021)) are committed to providing a virtual link between the digital workplace and the physical environment by means of a Cloud-based platform able to collect users data. These data might also be used to monitor user activity [85] or even suggest the most appropriate time intervals to take a break [86] considering the user’s focus state. Collected data can also come from a smart chair that could be used to improve the user’s sitting position [87]. Novel technologies such as 5G in IoT domains have been devised to boost comfort [88] and safety [89] in working environments.

As far as energy awareness in working environments is concerned, there have also been some proposals so far. For instance, a digital interface was proposed by Irizar-Arrieta et al. [90] that was aimed to notify users about their associated energy consumption. This is very similar to the interactive coaster developed in the context of the GS project (Section 2.2) which was aimed to make workers aware of the energy consumption of the electronic devices that were naturally spread over their offices [91]. Recently, there have been proposals aimed at reaching a large number of users: from displaying statistics in real-time regarding energy consumption in a physical ground of a factory [92] to measuring the power consumption of shared laboratory equipment [93], including proposals to transform working tools and equipment into smart devices that persuade their users with eco-awareness [26].

Moreover, some works have already explored the human factor behind these interventions and how people and the devices that populate smart workplaces can cooperate towards higher energy efficiency [94] or bringing health awareness to the workplace by increasing technology acceptance [95]. In general, work environments are especially challenging scenarios where additional barriers regarding privacy concerns of the collected information [96] and the ethical concerns [58] must be considered. Moreover, context and commitment to change are also a key factor when workday duties involve the total daily routine [97].

This work goes one step further in the line of converting work environments into appropriate settings to promote the adoption of lifestyle changes that persist over time. In contrast to the literature reviewed, our proposal puts the focus on the users’ concerns as a way to successfully tailor their future actions. To that end, we present the requirements to design an open novel architecture able to allocate interactive interventions in the workplaces while considering system scalability, users’ privacy and cost. Moreover, this work highlights the role of the worker at the center of a system that addresses both energy consumption and workers health, as a whole rather than tackling these aspects individually with expensive or commercial (e.g., Comfy Enlighted (available online: https://www.enlightedinc.com/ (accessed on 7 May 2021)) ad hoc single purpose devices.

In essence, the presented approach links innovative data architectures with the future work environment while addressing the human role in the process.

## 7. Conclusions

The IoT paradigm has enabled the rapid conception of a plethora of new applications and use-cases committed to improving and supporting humans’ daily lives. However, despite the apparent benefits brought by these solutions, there is a growing number of users who exhibit a somehow averse behavior towards these improvements. In this work, we describe and analyze two IoT use-cases (i.e., Smart Sustainable Coffee Machines and GreenSoul projects) to identify the source of these reluctant attitudes and set up the grounds of an architecture to address them. The results from both tested deployments allow us to conclude the importance of involving users to take actions in the smart environment themselves while preserving their privacy preferences. This motivated the design of PyFF, a privacy-friendly by design architecture aimed to enable the transformation of physical spaces into smart environments by actively involving the user in such a process.

PyFF is a Privacy Fog-based Flexible approach where the user decides which data he or she wants to disclose (i.e., respecting privacy) and to what extent (i.e., exploiting the Fog and Cloud Computing paradigms). From these premises, PyFF can continuously monitor users’ activities and their environment and advise on the best actions to increase their comfort while, for instance, optimizing energy usage (i.e., through flexible ICT communication channels). Additionally, instead of conceiving expensive and new ad hoc gadgets, PyFF aims to take advantage of the off-the-shelf technology already deployed in user environments (e.g., desktop computers and smartphones) to sense the environmental status and user dynamics and naturally interact with them. To overcome the data storage and computing limitations associated to this continuous monitoring, PyFF features a Fog Computing domain (i.e., Early Stage Computing Layer) composed of all the digital devices deployed around the user (that can join or leave at will) and a Cloud Computing layer (i.e., intensive computing layer) that will be used whenever these devices need to carry more complex computations. Therefore, the combination of Fog and Cloud Computing layers enable PyFF to limit the scope of the sensed data according to the users’ preferences in relation to the privacy they wanted to preserve, while obscuring its data when needed (i.e., splitting the computation process in several distributed nodes improves data security [98,99]). In essence, other architectures [54] are focused on how to distribute the data, which data models to use, how many vendor protocols are able to endow or what means of interoperability are the most appropriate to define a minimum interoperable system (available online: https://oascities.org/minimal-interoperability-mechanisms/ (accessed on 7 May 2021)). However, PyFF has not yet proposed another architecture with more or fewer layers than others, but a way of understanding the data flow and the deployment based on the user requirements, needs and privacy concerns.

The conducted qualitative evaluation shows at what level PyFF can adjust its architecture to make it more flexible compared to both use-cases in terms of privacy, deployment cost and automation. The next steps for this research work are: (1) conduct experiments in a real environment to assess quantitative metrics; (2) deepen the security protocols to enhance the proposed privacy scheme; or (3) study the possibility of splitting each layer into micro services to offer more flexibility in terms of fault tolerance, heterogeneity and accuracy.

## Figures and Tables

**Table 1:** PyFF qualitative evaluation.

Metrics		Qualitative Evaluation		
		GreenSoul	Smart Sustainable Coffee Machines	PyFF
Privacy	Data protection	+ (anonymization & encryption)	+ (anonymization)	++ (based on privacy policy)
	Data usage	Edge	Cloud	Device, Edge, Cloud (based on user’s choice)
	Homogeneity	Yes	Yes	heterogenous privacy rules & preferences
	Disruption/Intrusion	-(many new deployed devices)	- -(full automation)	++(Interaction-based scheme & no extra devices)
Automation	User involvement	+(one-way recommendations)	- -(full automation)	++ (full-duplex & adapted to user involvement preferences)
	Recommendation accuracy	Fog-based	Cloud-based	Cloud/Fog (parameter)
	ICT/HCI	dashboard	dashboard	depends on user’s behavior/preference
	Real-time	Yes	Yes	Yes
Flexibility	Adaptive reasoning	Non-existent	Non-existent	layer-based
	Context-based	Energy	Energy(coffee machines)	Any context
	Scalability	workplace -	home & workplace +	++
Deployment	Deployment cost	Hardware + software	Hardware + software	Hardware + software
	Fault isolation and tolerance	NA	Yes	Yes
	Heterogeneous devices	Yes	No	Yes
	Reliability	- (fog-ML-based recommendation)	+(Statistical method)	NA
	Distributed	No	No	Yes
	Event management	+ DSS	NA	++ DSS + User-Environment layer
